# Report of a case with T1a gallbladder poorly differentiated adenocarcinoma, solid type, which developed into lymph node metastases

**DOI:** 10.1186/s40792-015-0117-2

**Published:** 2015-11-16

**Authors:** Atsushi Takano, Shota Harai, Hiroshi Nakagomi, Masahiro Maruyama, Atsushi Yamamoto, Hideki Watanabe, Haruka Nakada, Kazushige Furuya, Masao Hada, Yoshiaki Miyasaka, Toshio Oyama, Masao Omata

**Affiliations:** Department of Surgery, Yamanashi, Japan; Department of Pathology, Yamanashi, Japan; Department of Digestive Medicine, Yamanashi, Japan; University of Tokyo, Tokyo, Japan; 1-1-1 Fujimi Kofu, Yamanashi, 400-8506 Japan

**Keywords:** T1a gallbladder carcinoma, Lymph node metastases, Lymphatic vessels

## Abstract

We experienced a case with gallbladder carcinoma growing limited to the mucosa (T1a), which developed massive lymphatic vessel spread and lymph node metastases.

A 72-year-old man was referred to our hospital for the swelling of his gallbladder during a routine ultrasound sonography checkup. We diagnosed the patient with gallbladder carcinoma with lymph node metastasis according to the radiographic findings and performed the open cholecystectomy and lymph node dissection. A histological examination showed poorly differentiated adenocarcinoma, solid type, and the tumor was limited to the mucosa. The number of lymphatic vessels was increased in the tumor and peritumor areas, and cancer cells were observed in the lymphatic vessels, which were detected via D2-40 immunohistochemistry. A careful histological examination and follow-up is required for T1a gallbladder carcinoma.

## Background

The prognosis of gallbladder carcinoma is poor. The 5-year survival rate of surgical resection was reported to be 40 % [1]. However, patients with T1a gallbladder carcinoma (GC) are considered to be curable by cholecystectomy without lymph node dissection [2, 3], and no evidence has shown that the lymph node dissection improves the prognosis of T1a GC [4–6]. Furthermore, the frequency of lymph node metastases of T1a GC was reported to be 0–2.5 % [6, 7].

However, we experienced a case with gallbladder carcinoma growing limited in the mucosa developed massive lymphatic vessel spread and lymph node metastases.

We herein report the rare case of lymph node metastases in a patient with GC and discuss the considerations for the etiology of lymph node metastases in this case.

## Case presentation

A 72-year-old man was referred to our hospital for the swelling of his gallbladder which was indicated during a routine ultrasound sonography checkup. He had been previously treated for hypertension, diabetes mellitus, and a cerebral infarction. He had no family history of cancer. There were no physical abnormalities on this admission. The laboratory data indicated a slight elevation of γ-GTP at 119 IU/l, but no other abnormal findings. The values of tumor markers were within the normal ranges; CEA, 2.6 ng/ml and CA19-9, 29.3 U/ml.

**Fig. 1:**
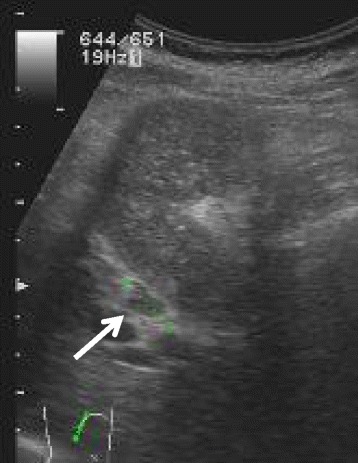
Ultrasound sonography showed enlarged gallbladder filled with concentrated bile juice and enlarged lymph node along the GB wall. The wall thickness was not detected

**Fig. 2:**
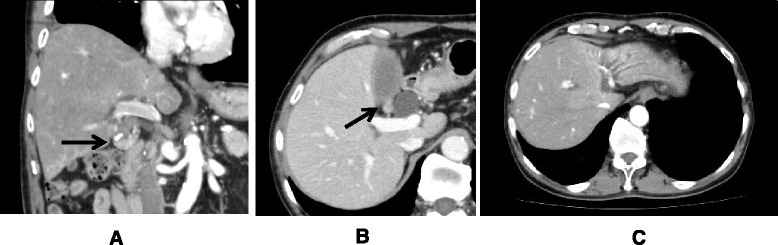
Coronal section of computed tomography indicated contrast-enhanced wall thickness of the neck of the gall bladder (**a**) and axial section showed swelling of lymph node measuring 1.2 cm in diameter (**b**). The dilated intrahepatic bile duct was seen in left liver (**c**); this finding was consistently observed in the patient’s clinical course and indicated no malignant findings

Ultrasound sonography showed an enlarged gallbladder filled with concentrated bile juice and an enlarged lymph node along the GB wall. The wall thickness was not detected (Fig. 1). Computed tomography indicated a contrast-enhanced wall thickness of the neck of gall bladder and swelling of the lymph node measuring 1.2 cm in diameter (Fig. 2).

**Fig. 3:**
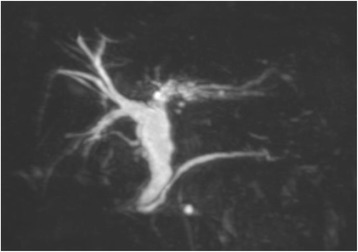
MRI showed dilated extra hepatic bile duct and intrahepatic bile duct in left liver and no finding of anomalous connection with pancreatic duct

MRI showed dilated common bile duct at 18 mm in diameter and no finding of the anomalous connection with the pancreatic duct (Fig. 3). Although, the dilated intrahepatic bile duct was seen in left liver; this finding was consistently observed in the patient’s clinical course and indicated no malignant disease.

According to these findings, we diagnosed the patient with gallbladder carcinoma with lymph node metastasis and performed open cholecystectomy and lymph node dissection of the hepatoduodenal ligament. An analysis of intraoperative frozen sections revealed tumor invasion to the cystic duct; therefore, we additionally resected the extrahepatic bile duct and regional lymph nodes. The concentration of biliary amylase was not elevated at 196 IU/l.

**Fig. 4:**
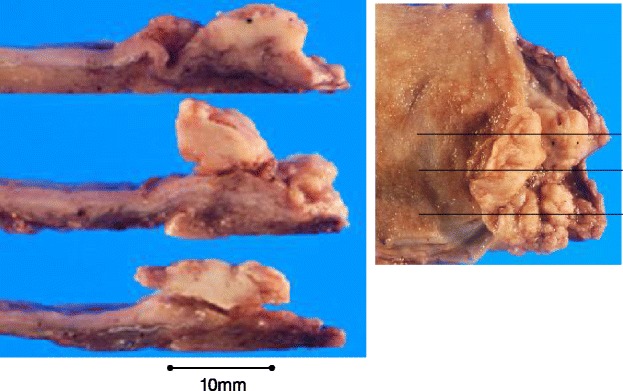
The gross appearance showed a papillary-expanding tumor at the neck of gallbladder

**Fig. 5:**
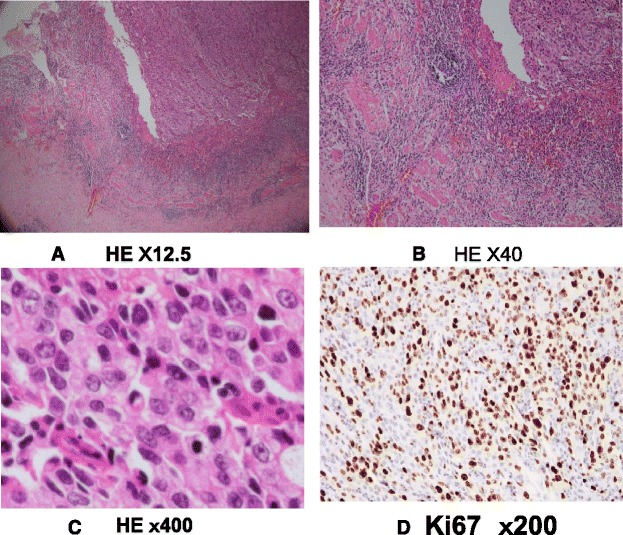
A histological finding showed cancer was growing limited to the mucosa (**a** and **b**). According to the no tubular formation and high nuclear grade, it was diagnosed poorly differentiated adenocarcinoma, solid type (**c**). Ki 67 labeling index was high value at 70–80 % (**d**)

**Fig. 6:**
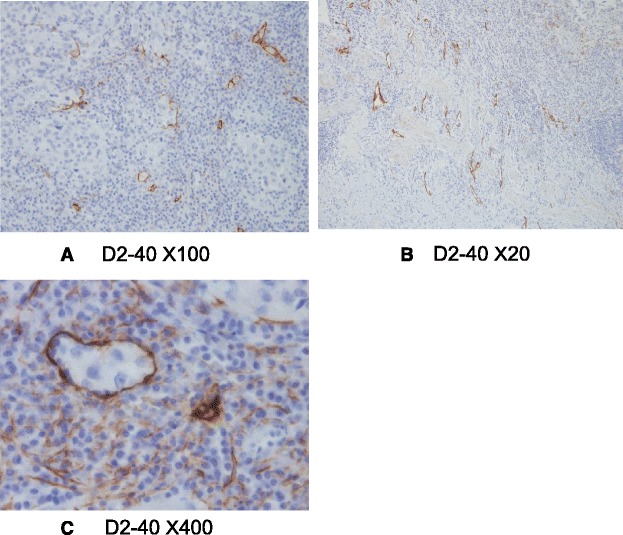
D2-40 immunohistochemistry; The number of lymphatic vessel was increased in tumor (**a**) and peritumor areas (**b**), and cancer cells were observed in the lymphatic vessels (**c**)

The gross appearance showed a papillary-expanding tumor at the neck of the gallbladder (Fig. 4) and the swelling of the lymph node. A histological examination showed poorly differentiated adenocarcinoma, solid type, and the tumor was limited to the mucosa (Fig. 5). The number of lymphatic vessels was increased in the tumor and peritumor areas, and cancer cells were observed in the lymphatic vessels, which were detected via D2-40 immunohistochemistry (Fig. 6). The clinical and pathological findings were summarized as GnC, papillary-expanding type, circ, 20 × 15mm, por1, med, INFβ, pHinf0, pBinf0, pPV0, ly (1), v0, pn0, s(−), pT1a (M), pN1(2/4) (12c(1/1), 12p(1/2) 12a(0/1)), DM(0), HM (0), EM (0), according to General Rules for Clinical and Pathological Studies on Cancer of the Biliary Tract. 6th Edition [8].

Although a frozen section of the cystic duct indicated tumor invasion, the tumor invasion into the distal bile duct and perineural invasion were not seen.

The patient was administered gemcitabine (1000 mg/m2/biweekly) and S1 (100 mg/every other day) as an adjuvant therapy. However, lymph node metastases occurred in the paraaortic and supraclavicular regions, 10 months after the operation. We subsequently added radiation therapy for both regions and continued gemcitabine and S1 for 2 years.

### Discussion

Lymph node metastasis is one of the most important prognostic factors of gallbladder carcinoma [9], as well as the depth of tumor invasion, histological grade, and perineural invasion [5, 10, 11].

The frequency of lymph node metastases by tumor invasion is as follows: T1a, the tumor invaded into the mucosa 0–2.5 %; T1b, the tumor invaded into muscularis 5–16 %; T2, the tumor invaded into the perimuscular connective tissue 9–30 %; T3, the tumor perforated into the serosa 39–72 %; and T4, directly invaded into other organs 67–80 % [7].

The wall of the gallbladder is composed of three layers: mucosa, muscularis, and serosa. There is no muscularis mucosa or submucosa [12]. Tumors in the mucosa easily invade the muscular layer and subserosa. The specific structure of the gallbladder wall might be one of the reasons why T1a GC led to lymph node metastases.

Even in incidental gallbladder carcinoma, T2 (67 %) and T3 (25 %) diseases are dominant [13]. Only approximately 20 % of patients with incidentally diagnosed GC have early stage disease [13].

The histological grade is strongly related to the prognosis of gallbladder carcinoma. The frequency of lymph node metastases is higher in high nuclear grade tumors or poorly differentiated adenocarcinomas [10].

The specific histological finding in the present case was that lymphatic vessels were observed in the tumor. This finding suggests that cytokines are produced in the tumor tissues which induced the lymphatic vessels. Recent reports have indicated that vascular endothelial growth factor C and D induced neoplastic lymph angiogenesis and were related to lymph node metastasis in several cancers, including gallbladder carcinoma [14–16].

Because a lymph node metastasis was suspected according to the preoperative evaluations in this case, we initially diagnosed the patient with T2 gallbladder carcinoma and performed lymph node dissection. If a case would be incidentally diagnosed as T1a gallbladder carcinoma, the additional lymph node dissection was not performed according to the guidelines. 

## Conclusions

We have experienced a case with T1a gallbladder carcinoma developed lymph node metastases. Therfore, even in T1a gallbladder carcinoma, a careful histological examination and follow-up is required.

## Consent

Written informed consent was obtained from the patient for publication of this case and any accompanying images. A copy of the written consent is available for review by the Editorial-in-Chief of this journal.
